# Reduced T and NK Cell Activity in Glioblastoma Patients Correlates with TIM-3 and BAT3 Dysregulation

**DOI:** 10.3390/cells13211777

**Published:** 2024-10-26

**Authors:** Farah Ahmady, Peter Curpen, Louis Perriman, Adilson Fonseca Teixeira, Siqi Wu, Hong-Jian Zhu, Arpita Poddar, Aparna Jayachandran, George Kannourakis, Rodney B. Luwor

**Affiliations:** 1 Fiona Elsey Cancer Research Institute, Ballarat, VIC 3350, Australia; farah@fecri.org.au (F.A.); louis.perriman@mcri.edu.au (L.P.); arpita@fecri.org.au (A.P.); aparna@fecri.org.au (A.J.); george@fecri.org.au (G.K.); 2 Federation University, Ballarat, VIC 3350, Australia; 3 Townsville Hospital and Health Service, James Cook University, Townsville, QLD 4814, Australia; jensencurpen@outlook.com; 4 Murdoch Children’s Research Institute, Parkville, VIC 3052, Australia; 5 Department of Surgery, The Royal Melbourne Hospital, The University of Melbourne, Parkville, VIC 3050, Australia; afonsecateix@student.unimelb.edu.au (A.F.T.); wus8@student.unimelb.edu.au (S.W.); hongjian@unimelb.edu.au (H.-J.Z.); 6 Huagene Institute, Kecheng Science and Technology Park, Pukou District, Nanjing 211806, China

**Keywords:** TIM-3, BAT3, glioblastoma, T cell exhaustion

## Abstract

Inhibitory receptors are critical for regulating immune cell function. In cancer, these receptors are often over-expressed on the cell surface of T and NK cells, leading to reduced anti-tumor activity. Here, through the analysis of 11 commonly studied checkpoint and inhibitory receptors, we discern that only *HAVCR2* (TIM3) and *ENTPD1* (CD39) display significantly greater gene expression in glioblastoma compared to normal brain and lower grade glioma. Cell surface TIM-3, but not ENTPD1, was also elevated on activated CD4^+^ and CD8^+^ T cells, as well as on NK cells from glioblastoma patients compared to healthy donor T and NK cells. A subsequent analysis of molecules known to co-ordinate TIM-3 function and regulation was performed, which revealed that BAT3 expression was significantly reduced in CD4^+^ and CD8^+^ T cells, as well as NK cells from glioblastoma patients compared to counterparts from healthy donors. These pro-inhibitory changes are also correlated with reduced levels of the activation marker CD69 and the pro-inflammatory cytokine IFNγ in CD4^+^ and CD8^+^ T cells, as well as NK cells from glioblastoma patients. Collectively, these data reveal that glioblastoma-mediated CD4^+^ and CD8^+^ T cell and NK cell suppression is due, at least in part, to dysregulated TIM-3 and BAT3 expression and the associated downstream immunoregulatory and dysfunctional effects.

## 1. Introduction

Glioblastoma is the most frequent and aggressive malignant brain tumor, with a median overall survival of 12–15 months after diagnosis [1,2,3]. Despite advances in identifying novel genetic alterations and therapeutic targets in glioblastoma, as well as trialing immunotherapeutic strategies that have been successful in other tumor types, the standard of care and overall survival of glioblastoma patients has not changed in almost two decades [4,5,6,7]. This poor survival rate is due in part to the pro-immunosuppressive characteristics of the glioblastoma tumor micro-environment, leading to the absence of or reductions in T and natural killer (NK) cells’ anti-tumor responses [8,9]. The molecular mechanisms of such T and NK cell inhibition are not well understood and are likely due to several factors, including the dysregulation and subsequent over-expression of checkpoint or inhibitory receptors, or changes in the cytokine milieu that favor a T and NK cell exhausted phenotype and an overall immunosuppressive tumor micro-environment [10]. Therefore, blocking checkpoint or inhibitory receptor function is a rational therapeutic approach for improving clinical outcomes in the glioblastoma setting. 

One immunoregulatory molecule that has not been thoroughly targeted in glioblastoma-based clinical trials is T cell immunoglobulin and mucin domain-containing protein 3 (TIM-3, encoded by the *HAVCR2* gene). TIM-3, along with TIM-1 and TIM-4, are members of the TIM family that all play roles in regulating immune function [11]. TIM-3 was first identified on Interferon-γ (IFNγ)-secreting effector CD4^+^ and CD8^+^ T cells [12,13]. Subsequently, TIM-3 expression has been identified as a key inhibitory receptor and has been observed on several other immune cell types including regulatory T (Treg) cells, NK cells, dendritic cells, and macrophages [14,15,16]. TIM-3 binds four known ligands: galectin-9, high mobility group protein B1 (HMGB1), carcinoembryonic antigen cell adhesion molecule 1 (CEACAM1), and phosphatidylserine [14,17,18,19]. Ligand-free TIM-3 is bound by HLA-B-associated transcript 3 (BAT3, also known as BAG6) at the cytoplasmic tail of TIM-3, rendering it permissive and allowing for enhanced T cell activity and cytotoxicity, enhanced secretion of inflammatory cytokines (such as IFNγ), and reduced T cell terminal differentiation and exhaustion [20,21,22]. Conversely, it has been reported that when TIM-3 is bound to a ligand, a sequential cascade of signaling events occurs, including TIM-3 phosphorylation; BAT3 disassociation; increased phosphorylation and the subsequent nuclear displacement of the transcriptional repressor forkhead box protein O1 (FOXO1); and promotion of the expression and transcriptional activity of B-lymphocyte-induced maturation protein 1 (BLIMP1) (encoded by the *PRDM1* gene) [22,23]. BLIMP1 subsequently regulates a series of genes associated with enhanced T cell dysfunction and apoptosis [19,20,22]. In addition, the key transcription factor interferon regulator factor 4 (IRF4) plays a critical role in T cell anti-tumor immunity and has been shown to correlate with TIM-3 expression [24,25]. Despite the abundance of knowledge regarding TIM-3-regulated T cell immunity, very little is known regarding the potential interplay and regulation of TIM-3 by BAT3, FOXO1, BLIMP1, and IRF4 in the glioblastoma setting. 

Thus, we performed a mechanistically thorough analysis of TIM-3 and key TIM-3-associated molecules in T and NK cells from glioblastoma patients and healthy donor individuals. Here, we demonstrate that the ability to suppress the percentage of TIM-3 positivity following the activation of CD4^+^ and CD8^+^ T cells, as well as NK cells, from glioblastoma patients is significantly reduced, and that these cells display reduced BAT3, CD69, and IFNγ expression compared to healthy donors. Thus, TIM-3 and reduced immune cell activity represent a potential key immunosuppressive mechanism in the glioblastoma setting to promote tumor progression. 

## 2. Materials and Methods

### 2.1. Healthy Donor and Glioblastoma Patient Samples

This study involved the use of peripheral blood mononuclear cells (PBMCs) from 17 healthy donors and 46 newly diagnosed (pre-surgery and pre-treatment) glioblastoma patients. Patient characteristics are outlined in Supplementary Table S1. Samples were obtained from the Mark Hughes Foundation Brain Cancer Biobank at the University of Newcastle, New South Wales, Australia. PBMC isolation from healthy individuals was performed using fresh buffy coats (under biological resources agreement with Australian Red Cross Lifeblood, Parkville, Australia) that underwent Ficoll-Paque Plus centrifugation and were then stored in LN2 (−186 °C). Approval to use PBMCs from healthy donors and glioblastoma patients, as well as the accompanying clinical information, was granted by Grampians Health and St John of God Ballarat Hospital Human Research Ethics Committee (ERM project IDs 37521 and 69601).

### 2.2. Bulk Dataset and Single-Cell RNA Sequencing Data Analysis

The data utilized in this study were obtained from the Chinese Glioma Genome Atlas (CGGA; http://www.cgga.org.cn/; 20 November 2023), which includes a comprehensive dataset of nearly 2000 primary and recurrent glioma samples. Specifically, mRNA data from the CGGA database were employed to investigate gene expression differences across grade II, III, and IV glioma for the following genes: *HAVCR2*, *ENTPD1*, *PDCD1*, *CTLA4*, *LAG3*, *CD160*, *KLRG1*, *CD96*, *BTLA*, *2B4*, *TIGIT*, *LGALS9*, *HMGB1*, and *CEACAM1.* To identify pivotal prognostic genes, we used the GEPIA2 online tool (http://gepia2.cancer-pku.cn/#index; 18 November 2023, leveraging data from 163 glioblastoma (GBM) tissues and 207 non-tumor brain tissues from The Cancer Genome Atlas (TCGA) and the Adult Gentype-Tissue Expression (GTEx). This tool facilitated the validation of expression patterns for the hub genes identified in our study. Additionally, we compared gene expression profiles between normal brain parenchyma and glioblastoma, focusing on the aforementioned genes: *HAVCR2*, *ENTPD1*, *PDCD1*, *CTLA4*, *LAG3*, *CD160*, *KLRG1*, *CD96*, *BTLA*, *2B4*, *TIGIT*, *LGALS9*, *HMGB1*, and *CEACAM1.* The USCS Xena Browser (https://xenabrowser.net; accessed on 5 June 2024) [26] was used to access and analyze data from The Cancer Genome Atlas (TCGA) glioblastoma cohort [27,28]. The results reported here are based upon data generated by the TCGA Research Network (https://www.cancer.gov/tcga, accessed on 5 June 2024). Normalized RNA sequencing-based data were used to evaluate correlations between the *HAVCR2* and *ENTPD1* mRNA levels in glioblastoma samples. Clinical data were used to investigate correlations between the progression-free survival probability of glioblastoma patients and the *HAVCR2* mRNA levels in glioblastoma samples. 

Data corresponding to single-cell RNA sequencing (scRNA-seq) analysis were accessed and visualized using the Single Cell Portal (https://singlecell.broadinstitute.org/single_cell, accessed on 5 June 2024) from the Broad Institute of MIT and Harvard. The dataset previously published by Neftel et al. (2019) [29] was used to investigate the percentage of single cells expressing indicated genes and the mean expression of such genes across cell populations within the glioblastoma micro-environment. Only adult glioblastomas were selected for this analysis. The exclusion of pediatric glioblastomas resulted in the removal of 2193 cells that were filtered from the original dataset. 

### 2.3. Flow Cytometry

PBMCs from healthy donors and glioblastoma patients were thawed on ice and seeded into 96-well round bottom plates (Thermo Fisher Scientific, Waltham, MA, USA). Cells were then stimulated for 5 h with or without 5 ng/mL phorbol 12-myristate 13-acetate (PMA; Sigma, Castle Hill, NSW, Australia) and 0.75 µg/mL ionomycin (Sigma, Castle Hill, NSW, Australia). Cells were treated with Golgiplug™ (BD Bioscience, Franklin Lakes, NJ, USA) to inhibit protein secretion for the final 5 h prior to harvesting, and cells were then stained with antibodies for flow cytometry assessment. For galectin-9 and HMGB1 recombinant protein experiments, healthy donor PBMCs were defrosted and seeded into 96-well round bottom plates and stimulated with galectin-9 (0–2 μg/mL) or HMGB1 (0–2 μg/mL) 24 h prior to PMA and ionomycin stimulation. Cells were then stained with antibodies for flow cytometry assessment. 

Cells were incubated with a cocktail of the following conjugated antibodies: human cell surface antigen-specific antibodies (BD Biosciences: CD3-BV650 clone UCHT1 cat #563852; CD4-PercpCy5.5 clone SK3 cat #566923; CD4-PE-CF594 clone RPA-T4 cat #562281; CD8-Alexa Fluor 647 clone RPA-T8 cat #55708; CD8-BV510 clone RPA-T8 cat #563256; CD8-PE/Cy5 cat #555636; CD19-PE-Cy7 clone SJ25C1 cat #557835; CD25-BV711 clone 2A3 cat #563159; CD56-BV605 clone NCAM16.2 cat #562780; and CD127-BV421 clone HIL-7R-M21 cat #562437. TIM-3-BV786 clone 7D3 cat #568504; BioLegend: CD8 PE/Cy5 clone HIT8a cat #300910; CD19-PercpCy5.5 clone 6D5 cat #115534; and CD39-APC Fire 750 clone A1 cat #328230) were used to identify specific cell populations. The surface cocktail also included fixable viability stain 700 (BD Biosciences) to exclude dead cells and human Fc block (BD Biosciences). The cocktail was made using FACS buffer (1 × PBS with 0.1% BCS), and cells were stained for 15 min on ice in the dark. Cells were then fixed/permeabilized following surface staining and washing in FACS buffer using the BD cytofix/cytoperm kit (BD Biosciences), as per the manufacturer’s instructions. Cells were then washed in Perm/Wash buffer (BD cytofix/cytoperm kit) and subsequently stained with human-specific intracellular antibodies (BD Biosciences: BLIMP1-PE-CF594 clone 6D3 cat #565274; BioLegend: CD69-Alexa Fluor 488 clone HIB19 cat #557697; FOXO1-Alexa Fluor 647 clone W20064D cat #947206; IFNγ-PE clone 4S.B3 cat #502509; IRF4-Percp/Cy5.5 clone 34E cat #646416; and Abcam: BAT3-PE [EPR9223] cat #ab210838) in Perm/Wash buffer for 30 min on ice in the dark. Cells were then washed in Perm/Wash buffer and run through the flow cytometer in FACS buffer to detect surface and intracellular protein expression. All samples were run through a BD LSR Fortessa, and flow cytometry data were analyzed using FlowJo software (Treestar, V10.9.0). Analyses to determine statistical significance were conducted using GraphPad Prism (GraphPad 10.2.3 Software).

### 2.4. Statistical Analysis

Non-parametric tests were used for the statistical analysis of all flow cytometry experiments. A Mann–Whitney U test was used for comparisons between 2 groups and a Kruskal–Wallis test followed by a Dunn’s multiple comparisons test was used for comparisons between 3 groups. Statistical analysis of the gene expression data was performed using a one-way ANOVA.

## 3. Results

### 3.1. HAVCR2 and ENTPD1 Are Over-Expressed in Glioblastoma Tumors Micro-Environment

**Figure 1:**
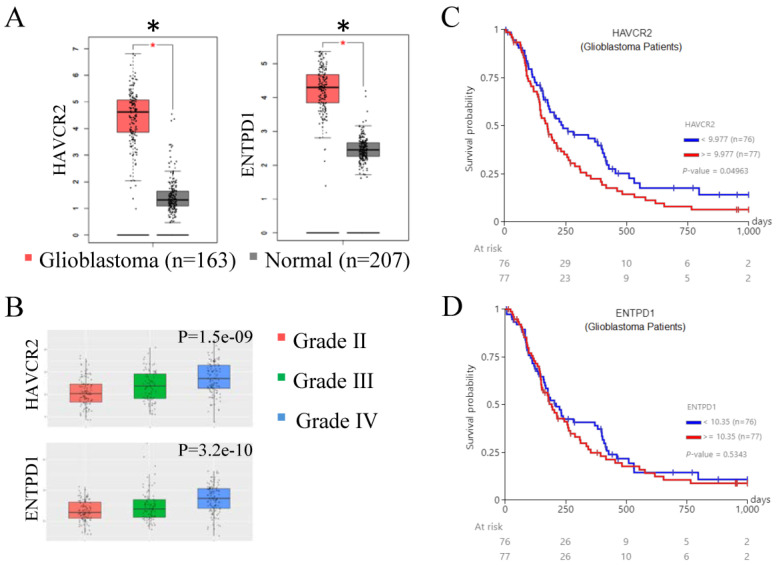
HAVCR2 and ENTPD1 are over-expressed in glioblastoma. (**A**) HAVCR2 and ENTPD1 gene expression levels in glioblastoma tumor tissue (red; n = 163) and normal brain tissue (grey; n = 207) were determined through mining the GEPIA2 TCGA dataset. (**B**) HAVCR2 and ENTPD1 gene expression levels in glioblastoma (grade IV) tumor tissue (blue), grade III glioma tumor tissue (green), and grade II glioma tumor tissue (red) were determined through mining the CGGA dataset. Kaplan–Meier curves showing the progression-free survival probability for glioblastoma patients, stratified for high gene expression (red line; n = 77) versus low gene expression (blue line; n = 76) of (**C**) HAVCR2 and (**D**) ENTPD1 mRNA levels analyzed in glioblastoma samples. Data were retrieved from the TCGA Glioblastoma cohort and analyzed using UCSC Xena. * *p* ≤ 0.05.

Several checkpoint and inhibitory receptors, such as PD-1 (gene name: *PDCD1*), CTLA4, TIM-3 (*HAVCR2*), LAG3, CD39 (*ENTPD1*), CD160, KLRG1, CD96, BTLA, 2B4, and TIGIT, have been implicated in the reduction of immune activity and response in the tumor micro-environment [30,31,32]. Therefore, we initially analyzed the expression levels of these 11 checkpoint and inhibitory receptors in glioblastoma tumors compared to normal brain tissue or grade II and III glioma using the GEPIA2 and CCGA databases. Our analysis found that significantly greater gene expressions of *HAVCR2* and *ENTPD1* (but not *PDCD1*, *CTLA4*, *LAG3*, *CD160*, *KLRG1*, *CD96*, *BTLA*, *2B4*, or *TIGIT*) were observed in glioblastoma compared to normal brain tissue (Figure 1A and Figure S1A), while *HAVCR2* and *ENTPD1* had the greatest significant differences between glioblastoma (grade IV) and grade II and III glioma of the 11 genes examined (Figure 1B and Figure S1B). Reinforcing these results, a strong and positive correlation was observed between *HAVCR2* and *ENTPD1* expression levels in glioblastoma patients (Supplementary Figure S1C). Moreover, elevated *HAVCR2* (but not *ENTPD1*) expression levels were significantly associated with reduced progression-free survival (PFS), but not overall survival for glioblastoma patients (Figure 1C,D). These results indicate an important contribution of checkpoint and inhibitory receptors in glioblastoma progression, especially *HAVCR2* (TIM-3). A significant difference in PFS for *HAVCR2* gene expression remains statistically significant if the stratification is by tertiles (*p* = 0.007) or quartiles (*p* = 0.06) (Supplementary Figure S1D). However, no significant difference has been observed in *ENTPD1* gene expression when separated into tertile or quartiles (Supplementary Figure S1E).

### 3.2. Percentage of TIM-3 Positivity Does Not Change After Stimulation of CD4^+^ T, CD8^+^ T, and NK Cells from Glioblastoma Patients

**Figure 2:**
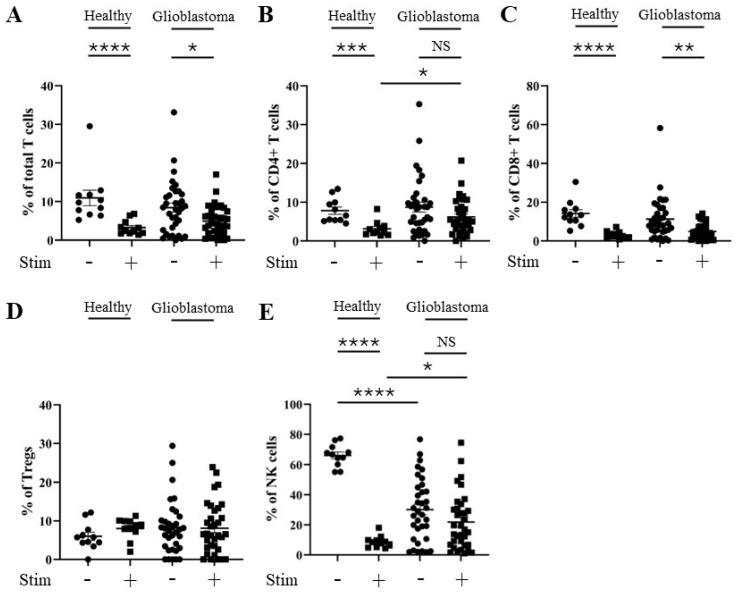
Percentage of TIM-3 positivity is similar for CD4^+^ T, CD8^+^ T, and NK cells from healthy control donors and glioblastoma patients. PBMCs from healthy controls and glioblastoma patients were treated with PMA, ionomycin, and Golgiplug™ for 5 h, then stained for surface TIM-3 prior to flow cytometry analysis. The unstimulated and stimulated percentages of TIM-3 positivity for each healthy control (n = 11) and glioblastoma patient (n = 35) are presented for (**A**) total T cells, (**B**) CD4^+^ T cells, (**C**) CD8^+^ T cells, (**D**) Tregs, and (**E**) NK cells. NS = not significant (>0.05); * *p* ≤ 0.05, ** *p* ≤ 0.01, *** *p* ≤ 0.001, **** *p* ≤ 0.0001.

**Figure 3:**
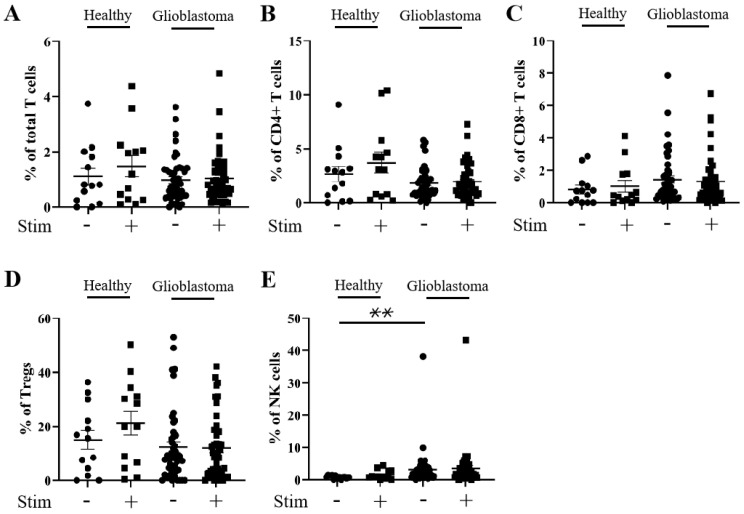
Percentage of CD39 positivity is similar for CD4^+^ T, CD8^+^ T, and NK cells from healthy control donors and glioblastoma patients. PBMCs from healthy controls and glioblastoma patients were treated with PMA, ionomycin, and Golgiplug™ for 5 h, then stained for surface CD39 prior to flow cytometry analysis. The unstimulated and stimulated percentages of CD39 positivity for each healthy control (n = 13) and glioblastoma patient (n = 46) are presented for (**A**) total T cells, (**B**) CD4^+^ T cells, (**C**) CD8^+^ T cells, (**D**) Tregs, and (**E**) NK cells. ** *p* ≤ 0.01.

As *HAVCR2* (TIM-3) and *ENTPD1* (CD39) gene expression levels were elevated in the glioblastoma micro-environment, we next compared the percentage positive levels of TIM-3 and CD39 on T and NK cells from glioblastoma versus healthy donor PBMCs ± stimulation with PMA and ionomycin using flow cytometry (patient and healthy control characteristics are listed in Supplementary Table S1). Specifically, we determined the percentage of TIM-3 and CD39 positivity on total T cells (CD3^+^), CD4^+^ T cells (CD3^+^; CD4^+^), CD8^+^ T cells (CD3^+^; CD8^+^), Tregs (CD3^+^; CD127^low^; CD25^+^), and NK cells (CD19^−^; CD3^−^; CD56^+^) (gating strategy outlined in Supplementary Figure S2). There was no significant increase in the percentage of TIM-3 positivity in any of the glioblastoma patient T cell subsets, and only a small increase in stimulated NK cells compared to healthy controls (Figure 2). However, a robust reduction in the percentage of TIM-3 positivity following the stimulation of healthy donors’ total T cells (reduced to 33% of unstimulated level), CD4^+^ T cells (reduced to 41% of unstimulated level), CD8^+^ T cells (reduced to 18% of unstimulated level), and NK cells (reduced to 13% of unstimulated level) (but not Tregs (increased to 149% of unstimulated level)) was observed. Stimulation with PMA and ionomycin had a greatly reduced effect on the percentage of TIM-3 positivity in glioblastoma patients’ total T cells (reduced to 63% of unstimulated level), CD4^+^ T cells (reduced to 80% of unstimulated level), CD8^+^ T cells (reduced to 46% of unstimulated level), Tregs (increased to 109% of unstimulated level), and NK cells (reduced to 81% of unstimulated level) (Figure 2A–E and Figure S3A–E). This suggests that, unlike those from healthy donors, T cell subsets and NK cells from glioblastoma patients may not achieve full activation through a maintained percentage of TIM-3 positivity. No difference was found in the level of CD39 positivity after stimulation in any of the T cell subsets or NK cells from healthy donors compared to those from glioblastoma patients (Figure 3A–E and Figure S3F–J). 

As we observed a reduction in the percentage of TIM-3 positivity after stimulation in T cell subsets and NK cells from glioblastoma patients, we determined whether the two ligands of TIM-3 (galectin-9 and HMGB1) that are significantly over-expressed in glioblastoma versus normal brain tissue and low-grade glioma (Supplementary Figure S4A–C) could modify the change in the percentage of TIM-3 positivity after stimulation in healthy donor CD4^+^ and CD8^+^ T cells and NK cells (Supplementary Figure S5A–C). Neither recombinant galectin-9 (Supplementary Figure S4C) nor recombinant HMGB1 (Supplementary Figure S4C) significantly modified the change in the percentage of TIM-3 positivity in these immune cell subsets from healthy donors after stimulation. 

### 3.3. Percentages of BAT3, FOXO1, and BLIMP1 Positivity Are Reduced in T and NK Cells from Glioblastoma Patients Versus Healthy Controls 

**Figure 4:**
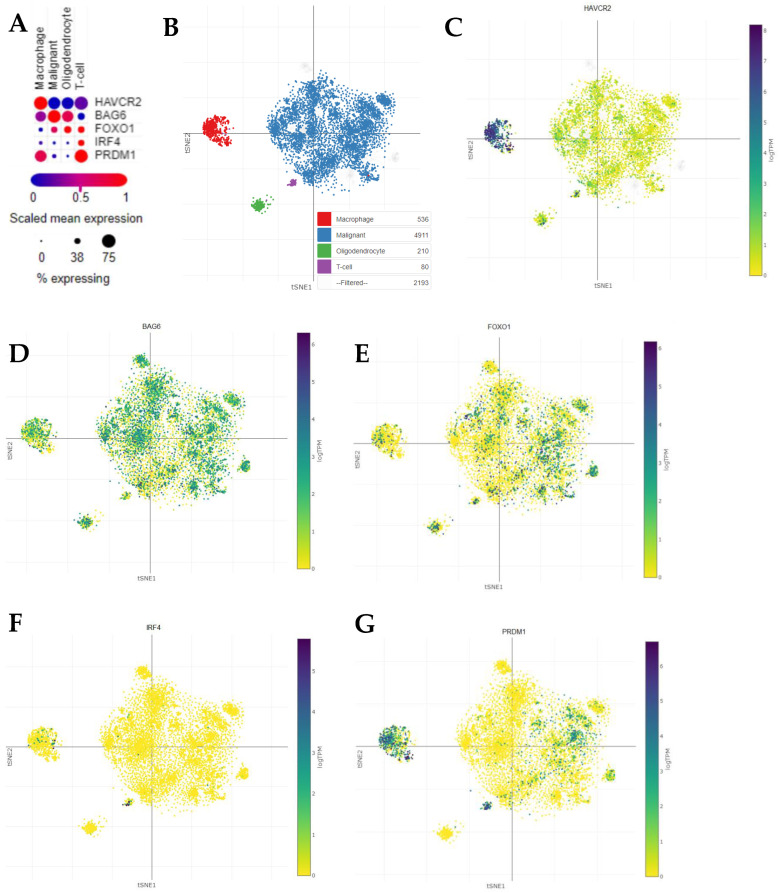
scRNA-seq analysis of glioblastomas reveals a large population of HAVCR2-expressing T cells. (**A**) A dot plot representing the mean expression of indicated genes and the percentage of macrophages, malignant cells, oligodendrocytes, and T cells expressing such genes in glioblastoma samples. (**B**) A t-distributed stochastic neighbor embedding (tSNE) plot of single cells identified in glioblastoma samples, as represented in (**A**), and colored according to cell type. tSNE plots for these cells are also presented and colored according to the mRNA levels quantified for (**C**) HAVCR2, (**D**) BAG6, (**E**) FOXO1, (**F**) IRF4, and (**G**) PRDM1. The dot plot and tSNE plots were generated using the Single Cell Portal (the Broad Institute of MIT and Harvard) by analyzing the scRNA-seq dataset published by Neftel et al. (2019) [29].

As TIM-3 expression has been linked to BAT3, FOXO1, IRF4, and BLIMP1 activity and expression, we next explored whether the expressions of these corresponding genes correlate with one another in the glioblastoma micro-environment. We analyzed a publicly available glioblastoma scRNA-seq dataset [29]. Interestingly, *HAVCR2* expression was detected in nearly 81% of the T cells analyzed, although at relatively low levels (Figure 4A–C). Moreover, different from malignant cells, macrophages, and oligodendrocytes, the detection of *HAVCR2* expression in most T cells was correlated with the reduced detection of T cells expressing *BAG6* (BAT3; Figure 4D), *FOXO1* (Figure 4E), and *IRF4* (Figure 4F). Conversely, *PRDM1* was strongly and broadly expressed by T cells (Figure 4G), as expected for downstream TIM-3 activation [21,22]. 

**Figure 5:**
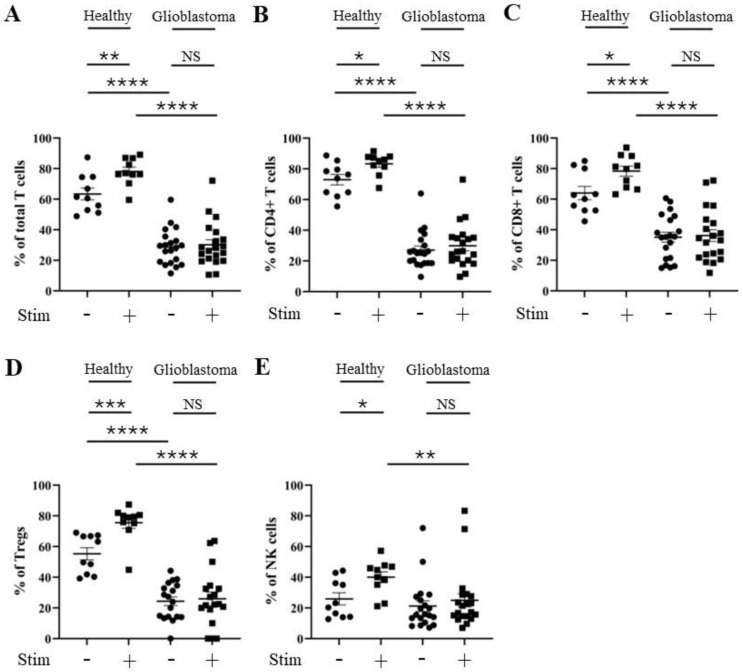
Percentage of BAT3 positivity is reduced in CD4^+^ T, CD8^+^ T, and NK cells from glioblastoma patients. PBMCs from healthy controls and glioblastoma patients were treated with PMA, ionomycin, and Golgiplug™ for 5 h, then stained for intracellular BAT3 prior to flow cytometry analysis. The unstimulated and stimulated percentages of BAT3 positivity for each healthy control (n = 10) and glioblastoma patient (n = 20) are presented for (**A**) total T cells, (**B**) CD4^+^ T cells, (**C**) CD8^+^ T cells, (**D**) Tregs cells, and (**E**) NK cells. NS = not significant (>0.05); * *p* ≤ 0.05, ** *p* ≤ 0.01, *** *p* ≤ 0.001, **** *p* ≤ 0.0001.

To further validate these findings, we investigated whether changes in the percentage positivity levels of BAT3, FOXO1, BLIMP1, and IRF4 intracellular proteins are observed in T and NK cells from glioblastoma patients compared to healthy controls. CD4^+^ and CD8^+^ T cells and NK cells from glioblastoma patients displayed significantly lower percentages of BAT3 positivity compared to those from healthy donors, irrespective of stimulation (Figure 5). Stimulation with PMA and ionomycin mediated a significant increase in the percentage of BAT3 positivity compared a lack of stimulation in total, CD4^+^, CD8^+^, Treg, and NK cells from healthy donors. However, we did not observe a significant increase in BAT3 positivity in stimulated T and NK cells from glioblastoma patients. This suggests that, unlike those from healthy donors, T cell subsets and NK cells from glioblastoma patients may not achieve full activation through a lower percentage of BAT3 positivity. 

**Figure 6:**
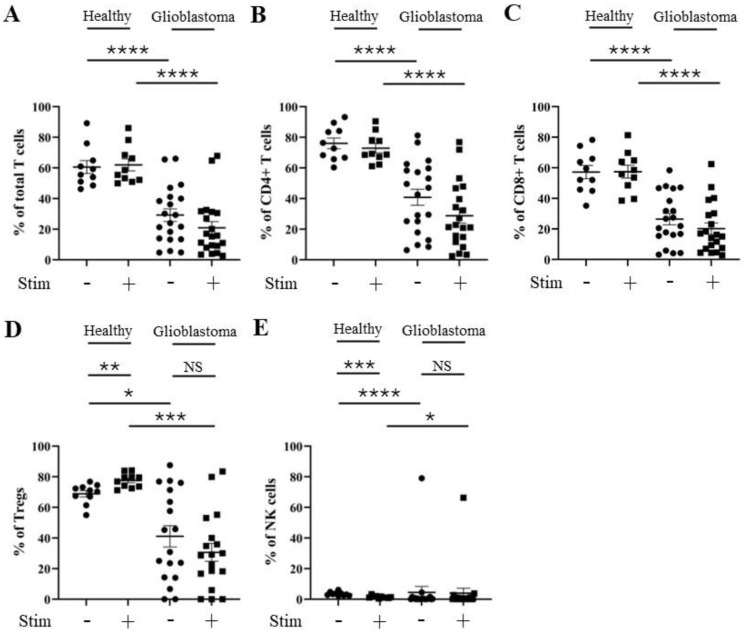
Percentage of FOXO1 positivity is reduced in CD4^+^ T, CD8^+^ T, and NK cells from glioblastoma patients. PBMCs from healthy controls and glioblastoma patients were treated with PMA, ionomycin, and Golgiplug™ for 5 h, then stained for intracellular FOXO1 prior to flow cytometry analysis. The unstimulated and stimulated percentages of FOXO1 positivity for each healthy control (n = 10) and glioblastoma patient (n = 20) are presented for (**A**) total T cells, (**B**) CD4^+^ T cells, (**C**) CD8^+^ T cells, (**D**) Tregs, and (**E**) NK cells. NS = not significant (>0.05); * *p* ≤ 0.05, ** *p* ≤ 0.01, *** *p* ≤ 0.001, **** *p* ≤ 0.0001.

**Figure 7:**
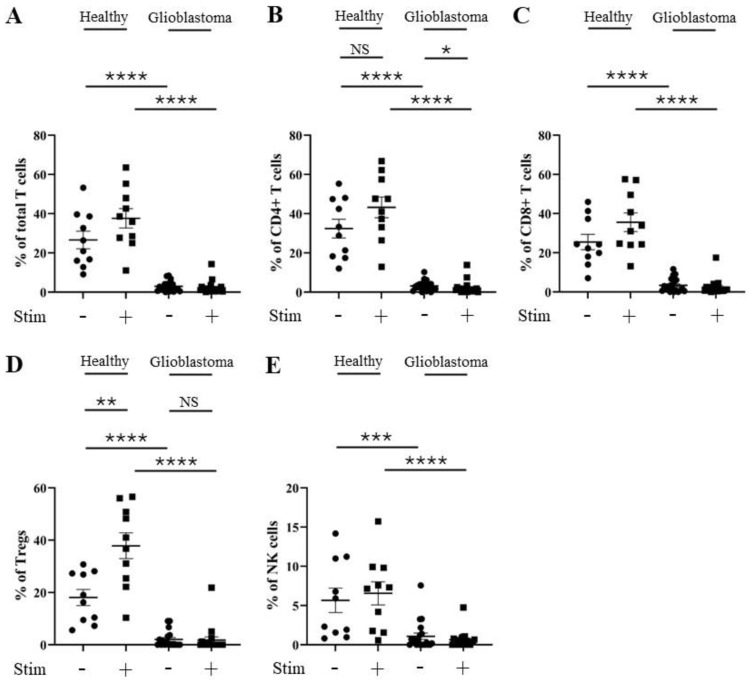
Percentage of BLIMP1 positivity is reduced in CD4^+^ T, CD8^+^ T, and NK cells from glioblastoma patients. PBMCs from healthy controls and glioblastoma patients were treated with PMA, ionomycin, and Golgiplug™ for 5 h, then stained for intracellular BLIMP1 prior to flow cytometry analysis. The unstimulated and stimulated percentages of BLIMP1 positivity for each healthy control (n = 10) and glioblastoma patient (n = 20) are presented for (**A**) total T cells, (**B**) CD4^+^ T cells, (**C**) CD8^+^ T cells, (**D**) Tregs, and (**E**) NK cells. NS = not significant (>0.05); * *p* ≤ 0.05, ** *p* ≤ 0.01, *** *p* ≤ 0.001, **** *p* ≤ 0.0001.

Similarly, the percentages of FOXO1 (Figure 6) and BLIMP1 (Figure 7) were also reduced in glioblastoma versus healthy donor CD4^+^ and CD8^+^ T cells and NK cells. No change was observed in IRF4 positivity (Supplementary Figure S5).

### 3.4. Percentages of CD69 and IFNγ Positivity Are Reduced in CD4^+^ T, CD8^+^ T, and NK Cells from Glioblastoma Patients Versus Healthy Controls 

**Figure 8:**
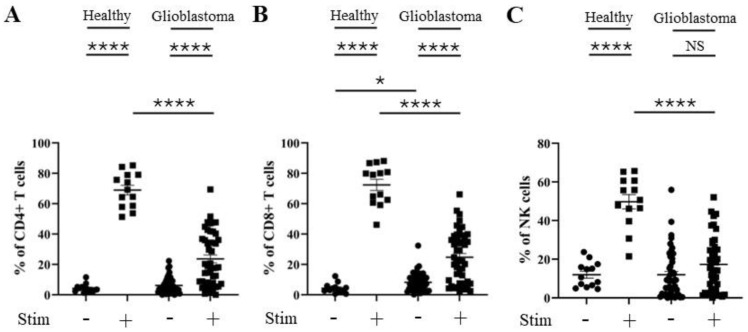
Percentage of CD69 positivity is reduced in CD4^+^ T, CD8^+^ T, and NK cells from glioblastoma patients. PBMCs from healthy controls and glioblastoma patients were treated with PMA, ionomycin, and Golgiplug™ for 5 h, then stained for intracellular CD69 prior to flow cytometry analysis. The unstimulated and stimulated percentages of CD69 positivity for each healthy control (n = 13) and glioblastoma patient (n = 46) are presented for (**A**) CD4^+^ T cells, (**B**) CD8^+^ T cells, and (**C**) NK cells. NS = not significant (>0.05); * *p* ≤ 0.05, **** *p* ≤ 0.0001.

As TIM-3 is associated with reduced T cell effector function, and as we observed changes in the percentage of TIM-3 positivity on CD4^+^ and CD8^+^ T cells and NK cells from glioblastoma patients, we next evaluated the activation statuses of these T cell subsets and NK cells. CD69 is a well-characterized activation marker for both T and NK cells [33,34]. The percentage of CD69 positivity was significantly elevated following PMA and ionomycin stimulation for CD4^+^ and CD8^+^ T cells and NK cells from healthy donors (Figure 8). However, a significantly reduced level of induction of CD69 percentage positivity was seen on stimulated CD4^+^ and CD8^+^ T cells and NK cells from glioblastoma patients compared to healthy donors (Figure 8). 

**Figure 9:**
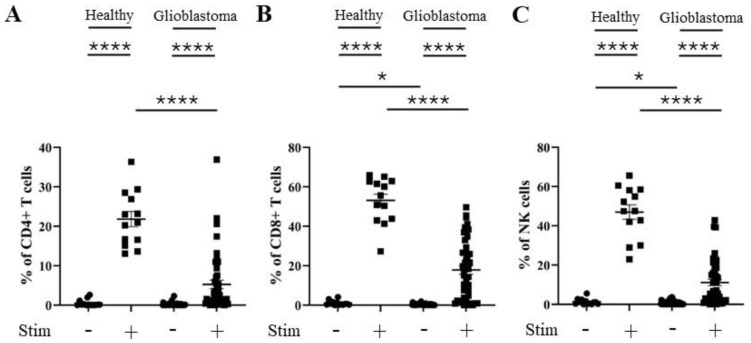
Percentage IFNγ positivity is reduced in CD4^+^ T, CD8^+^ T, and NK cells from glioblastoma patients. PBMCs from healthy controls and glioblastoma patients were treated with PMA, ionomycin, and Golgiplug™ for 5 h, then stained for intracellular IFNγ prior to flow cytometry analysis. The unstimulated and stimulated percentages of IFNγ positivity for each healthy control (n = 13) and glioblastoma patient (n = 46) are presented for (**A**) CD4^+^ T cells, (**B**) CD8^+^ T cells, and (**C**) NK cells. * *p* ≤ 0.05, **** *p* ≤ 0.0001.

In addition, similar differences were seen in the percentages of IFNγ positivity. IFNγ positivity was significantly increased when CD4^+^ and CD8^+^ T cells and NK cells from healthy donors and glioblastoma patients were stimulated with PMA and ionomycin (Figure 9). However, a significantly reduced level of induction of IFNγ positivity was seen on stimulated CD4^+^ and CD8^+^ T cells and NK cells from glioblastoma patients (Figure 9). 

## 4. Discussion

The function of TIM-3 as a key regulator of T cells is well recognized [35,36], with its over-expression implicated in glioblastoma progression [32,37,38,39], and our current data demonstrate that TIM-3 expression correlates with poor glioblastoma survival. However, the TIM-3-driven intracellular mechanisms that potentially lead to reduced T and NK cell activity in the glioblastoma setting have not been elucidated. Several conflicting reports have evaluated TIM-3 expression on CD4^+^ and CD8^+^ T cells in the glioblastoma setting [32,37,38,39]. Liu and colleagues demonstrated greater levels of TIM-3 expression on CD4^+^ and CD8^+^ T cells from glioma patients’ tissues (58% of which were glioblastoma) compared to healthy controls [38]. However, two subsequent reports using independent patient cohorts demonstrated no significant difference in TIM-3 expression on T cells from PBMC populations in glioblastoma patients compared to healthy donors [32,39]. These two previous studies displayed similar findings to ours, where unstimulated T and NK cells from glioblastoma patients did not exhibit enhanced percentages of TIM-3 positivity compared to those from healthy individuals. Indeed, unstimulated NK cells displayed lower percentages of TIM-3 positivity compared to matched healthy donor cells in our cohort of glioblastoma patients. However, unlike our current study, the work described above did not include data from stimulated T cells and, thus, did not explore potential differences when PBMCs were activated. Furthermore, these studies did not investigate potential upstream and downstream TIM-3-associated molecules or the consequences of elevated TIM-3. Our study incorporates a more thorough investigation of several potentially important molecules in T and NK cell activity. We examined the percentage of TIM-3 positivity and the activation status (via CD69 and IFNγ) following the PMA and ionomycin stimulation of glioblastoma patients’ T and NK cells compared to those of healthy individuals. Importantly, unlike CD39 (*ENTPD1*), another inhibitory marker shown to be elevated globally during gene expression analysis of glioblastoma tumor tissue, we demonstrated a significant inability of PMA and ionomycin stimulation to reduce TIM-3 on CD4^+^ and CD8^+^ T cells, as well as NK cells (but not Tregs), from glioblastoma patients. This reduction, as expected, was seen in healthy donor T and NK cells, leading to expected enhanced activation. This suggests that in glioblastoma patients, these cells lack the ability to be fully activated when primed, as evidenced by the reduced levels of IFNγ and CD69, which allows speculation that these cells may be terminally exhausted. Indeed, TIM-3 expression is regarded as a marker for terminally differentiated effector and exhausted cells [12], thereby suggesting that CD4^+^ and CD8^+^ T cells and NK cells may be terminally exhausted in glioblastoma patients. 

We also undertook a thorough mechanistic analysis of upstream and downstream substrates of TIM-3 (BAT3, FOXO1, BLIMP1, and IRF4). The analysis of BAT3 expression and function has not been extensively explored in cancer. Here, we demonstrate that the percentage of intracellular BAT3 positivity is reduced in CD4^+^ and CD8^+^ T cells and NK cells from glioblastoma patients. BAT3 deficiency in mouse T cells has led to enhanced TIM-3 and reduced IFNγ expression, causing an overall reduced T cell effector response and an enhanced T cell exhaustion phenotype [20]. Similarly, our subsequent analysis of single-cell RNA-Seq data from Neftel and colleagues [29] revealed that *HAVCR2* (TIM-3) expression was detected in most T cells (81%) in the glioblastoma micro-environment and was correlated with low expression of BAG6 (BAT3). These changes are likely due to the reduced association of BAT3 with the cytoplasmic tail of TIM-3, thereby allowing TIM-3 to promote T cell inactivity [22]. Although the number of assessable CD4^+^ and CD8^+^ T cells in each healthy donor and glioblastoma patient prevented us from examining changes in direct TIM-3:BAT3 interaction, our data are consistent with these reports, suggesting that percentage of TIM-3 positivity and downstream function is enhanced due to less BAT3 expression (and presumably reduced TIM-3 association). Importantly, our data also show that stimulation caused differential BAT3 changes in CD4^+^ T, CD8^+^ T, and NK cells from healthy donors compared to glioblastoma patients. In healthy donors, stimulation led to a significant increase in the percentage positivity of BAT3. This increased BAT3 positivity potentially favors greater TIM-3:BAT3 association and subsequently reduces TIM-3 expression and TIM-3-mediated T and NK cell inactivation. However, the stimulation of CD4^+^ T, CD8^+^ T, and NK cells from glioblastoma patients did not cause any change in BAT3 positivity. This further supports our notion that TIM-3 mediates immunosuppression via CD4^+^ T, CD8^+^ T, and NK cell inactivity through a TIM-3/BAT3 dysregulated mechanism. Although TIM-3 has been recognized as a key suppressive marker in NK function [40], very little is known regarding BAT3’s association with and/or regulation of TIM-3 in NK cells in cancer. Importantly, we also demonstrate here that there is a potential inverse correlation between the percentage of TIM-3 and BAT3 positivity and the subsequent reduced activity in NK cells from glioblastoma patients. This suggests that BAT3 may have a similar involvement with TIM-3 in NK cells, which belong to the innate arm of immunity, as it does in T cells, which belong to the adaptive arm of immunity (at least in the glioblastoma setting). 

Genetic knockdown of BAT3 in mouse T cells also triggers an increase in FOXO1 phosphorylation and an increase in the expression and transcriptional activity of a key regulator of T cell dysfunction, BLIMP1, through a TIM-3-dependent mechanism [22]. This enhanced BLIMP1 activity leads to reduced IFNγ expression and overall T cell activity. Conversely, our data demonstrate that CD4^+^ and CD8^+^ T cells from glioblastoma patients displayed significantly lower percentages of FOXO1 and BLIMP1 positivity compared to healthy controls. Although we did not examine the phosphorylated levels of FOXO1 or the transcriptional activity of BLIMP1 in T cells, we can assume, due to the very low percentage positivity of both FOXO1 and BLIMP1, that our data are dissimilar to that mentioned above, demonstrating that TIM-3/BAT3-mediated reduction of IFNγ expression and T cell dysfunction is likely dependent on FOXO1 phosphorylation and BLIMP1 transcriptional activity. We propose that there may be alternative TIM-3/BAT3 signaling-dependent mechanisms that converge to trigger reductions in CD69 and IFNγ, ultimately inhibiting CD4^+^ and CD8^+^ T cell activity without FOXO1 and BLIMP1 involvement (Graphical Abstract), although this is not fully elucidated in this current study. Similarly, the low abundance of CD4^+^ and CD8^+^ T cells and NK cells in the overall PBMC population from glioblastoma patients and, hence, the very low abundance of extracted RNA from these isolated populations prevented us from confirming flow results by qPCR. Nonetheless, overall we still identified dysfunctional levels of TIM-3 and BAT3 on CD4^+^ and CD8^+^ T cells and NK cells from glioblastoma patients.

Similarly, increased TIM-3 expression and decreased IFNγ expression were demonstrated in tumor-infiltrating CD8^+^ T cells in a syngeneic glioma mouse model, although potential changes in BAT3, FOXO1, BLIMP1, and CD69 expression were not reported in this study [32]. The potential TIM-3-dependent, FOXO1- and BLIMP1-independent mechanism of T cell dysfunction may also be evident in NK cells from glioblastoma patients, where the impaired reduction in the percentage of TIM-3 positivity after stimulation and the reduced percentage of BAT3 positivity are correlated with significant decreases in CD69 and IFNγ compared to control donors. However, a correlation between TIM-3 expression and NK cell exhaustion and dysfunction might be tumor type-specific. In accordance with our findings, TIM-3 expression on NK cells has been associated with reduced NK activity in both lung adenocarcinoma and melanoma settings [40,41], while TIM-3 levels have been correlated with enhanced IFNγ expression and NK cell cytotoxicity in acute myeloid leukemia [42]. To the best of our knowledge, our current study is the first to evaluate and identify significant reductions in the percentages of BAT3, CD69, and IFNγ positivity in CD4^+^ and CD8^+^ T cells and NK cells from glioblastoma patients.

The evidence suggests that galectin-9 promotes dissociation of BAT3 with TIM-3, subsequently promoting TIM-3-mediated T cell terminal differentiation, exhaustion, and cell death [20]. Indeed, glioma patients with galectin-9 over-expression have shown significantly shorter overall survival rates [43], while galectin-9/TIM-3 association mediates glioma development [44]. We did not observe any significant elevation in the percentage of TIM-3 positivity on healthy donor T and NK cells following short-term stimulation with galectin-9 (nor HMGB1, another known ligand for TIM-3). Interestingly, galectin-9 (and HMGB1) is elevated in glioblastoma tumor tissue, suggesting that persistent long-term stimulation with galectin-9 may be required for enhanced TIM-3 expression. Alternatively, whether another key molecule other than galectin-9 can directly or indirectly regulate TIM-3 expression in the glioblastoma setting is open for speculation and requires further study. 

Our current findings have potential clinical implications. Several clinical trials evaluating the PD-1 inhibitors Nivolumab and Pembrolizumab have produced disappointing outcomes and often failed to reach primary endpoints [7,45]. The lack of clinical efficacy of these inhibitors is attributed to several causes. However, one possibility is that the role of these inhibitory receptors may not be as critical for glioblastoma development compared to other tumor types. Other inhibitory and checkpoint receptors that have garnered less attraction for therapeutic targeting, such as TIM-3, may be more vital for overall tumor-driven immunosuppression in the glioblastoma setting, especially as TIM-3 (unlike many other checkpoint receptors) is over-expressed in glioblastoma tumor tissue and correlates with glioblastoma patients’ progression-free survival. Interestingly, *HAVCR2* gene expression is correlated with the progression-free, but not overall, survival of glioblastoma patients. As such, trialing TIM-3-targeting therapeutics may be more relevant in the current setting. Our results indicate that targeting TIM-3 directly may offer enhanced therapeutic benefit to those agents that target other checkpoint receptors. 

## 5. Conclusions

In summary, our data demonstrate the differential maintenance of TIM-3 surface expression on CD4^+^ and CD8^+^ T cells, as well as NK cells, from glioblastoma patients compared to cells from healthy donors following stimulation. This change correlates with a reduction in the positivity of BAT3, a key TIM-3-regulating protein. Unlike CD4^+^ and CD8^+^ T cells and NK cells from healthy donors, stimulation did not enhance the percentage positivity of BAT3 in CD4^+^ and CD8^+^ T cells or NK cells from glioblastoma patients. Subsequent reductions in the positivity of the cell activation marker CD69 and the key pro-inflammatory cytokine IFNγ were also observed in CD4^+^ and CD8^+^ T cells and NK cells from glioblastoma patients. Overall, our data indicate a novel mechanism of glioblastoma-induced suppression of T and NK cell effector and cytotoxic function, driven by TIM-3 and BAT3 dysregulation. 

## Data Availability

The data presented in this study are available upon direct request to the corresponding author.

## Supplementary Materials

The following supporting information can be downloaded at: https://www.mdpi.com/article/10.3390/cells13211777/s1.

## References

[1] Stupp R., Mason W.P., Bent M.J.v.D., Weller M., Fisher B., Taphoorn M.J., Belanger K., Brandes A.A., Marosi C., Bogdahn U. (2005). Radiotherapy plus Concomitant and Adjuvant Temozolomide for Glioblastoma. New Engl. J. Med..

[2] Louis D.N., Perry A., Reifenberger G., von Deimling A., Figarella-Branger D., Cavenee W.K., Ohgaki H., Wiestler O.D., Kleihues P., Ellison D.W. (2016). The 2016 World Health Organization Classification of Tumors of the Central Nervous System: A summary. Acta Neuropathol..

[3] Furnari F.B., Fenton T., Bachoo R.M., Mukasa A., Stommel J.M., Stegh A., Hahn W.C., Ligon K.L., Louis D.N., Brennan C. (2007). Malignant astrocytic glioma: Genetics, biology, and paths to treatment. Genes Dev..

[4] Farrell C., Shi W., Bodman A., Olson J.J. (2020). Congress of neurological surgeons systematic review and evidence-based guidelines update on the role of emerging developments in the management of newly diagnosed glioblastoma. J. Neuro-Oncology.

[5] McGranahan T., Therkelsen K.E., Ahmad S., Nagpal S. (2019). Current State of Immunotherapy for Treatment of Glioblastoma. Curr. Treat. Options Oncol..

[6] Ottaviano M., De Placido S., Ascierto P.A. (2019). Recent success and limitations of immune checkpoint inhibitors for cancer: A lesson from melanoma. Virchows Arch..

[7] Reardon D.A., Brandes A.A., Omuro A., Mulholland P., Lim M., Wick A., Baehring J., Ahluwalia M.S., Roth P., Bähr O. (2020). Effect of Nivolumab vs Bevacizumab in Patients with Recurrent Glioblastoma: The CheckMate 143 Phase 3 Randomized Clinical Trial. JAMA Oncol..

[8] Tomaszewski W., Sanchez-Perez L., Gajewski T.F., Sampson J.H. (2019). Brain Tumor Microenvironment and Host State: Implications for Immunotherapy. Clin. Cancer Res..

[9] Zhao J., Chen A.X., Gartrell R.D., Silverman A.M., Aparicio L., Chu T., Bordbar D., Shan D., Samanamud J., Mahajan A. (2019). Immune and genomic correlates of response to anti-PD-1 immunotherapy in glioblastoma. Nat. Med..

[10] Low J.T., Brown M.C., Reitman Z.J., Bernstock J.D., Markert J.M., Friedman G.K., Waitkus M.S., Bowie M.L., Ashley D.M. (2024). Understanding and therapeutically exploiting cGAS/STING signaling in glioblastoma. J. Clin. Investig..

[11] Meyers J.H., Sabatos C.A., Chakravarti S., Kuchroo V.K. (2005). The TIM gene family regulates autoimmune and allergic diseases. Trends Mol. Med..

[12] Wolf Y., Anderson A.C., Kuchroo V.K. (2019). TIM3 comes of age as an inhibitory receptor. Nat. Rev. Immunol..

[13] Monney L., Sabatos C.A., Gaglia J.L., Ryu A., Waldner H., Chernova T., Manning S., Greenfield E.A., Coyle A.J., Sobel R.A. (2002). Th1-specific cell surface protein Tim-3 regulates macrophage activation and severity of an autoimmune disease. Nature.

[14] Chiba S., Baghdadi M., Akiba H., Yoshiyama H., Kinoshita I., Dosaka-Akita H., Fujioka Y., Ohba Y., Gorman J.V., Colgan J.D. (2012). Tumor-infiltrating DCs suppress nucleic acid–mediated innate immune responses through interactions between the receptor TIM-3 and the alarmin HMGB1. Nat. Immunol..

[15] Ndhlovu L.C., Lopez-Vergès S., Barbour J.D., Jones R.B., Jha A.R., Long B.R., Schoeffler E.C., Fujita T., Nixon D.F., Lanier L.L. (2012). Tim-3 marks human natural killer cell maturation and suppresses cell-mediated cytotoxicity. Blood.

[16] Yan J., Zhang Y., Zhang J.-P., Liang J., Li L., Zheng L. (2013). Tim-3 Expression Defines Regulatory T Cells in Human Tumors. PLoS ONE.

[17] DeKruyff R.H., Bu X., Ballesteros A., Santiago C., Chim Y.-L.E., Lee H.-H., Karisola P., Pichavant M., Kaplan G.G., Umetsu D.T. (2010). T Cell/Transmembrane, Ig, and Mucin-3 Allelic Variants Differentially Recognize Phosphatidylserine and Mediate Phagocytosis of Apoptotic Cells. J. Immunol..

[18] Huang Y.-H., Zhu C., Kondo Y., Anderson A.C., Gandhi A., Russell A.F., Dougan S.K., Petersen B.-S., Melum E., Pertel T. (2015). CEACAM1 regulates TIM-3-mediated tolerance and exhaustion. Nature.

[19] Zhu C., Anderson A.C., Schubart A., Xiong H., Imitola J., Khoury S.J., Zheng X.X., Strom T.B., Kuchroo V.K. (2005). The Tim-3 ligand galectin-9 negatively regulates T helper type 1 immunity. Nat. Immunol..

[20] Rangachari M., Zhu C., Sakuishi K., Xiao S., Karman J., Chen A., Angin M., Wakeham A., A Greenfield E., A Sobel R. (2012). Bat3 promotes T cell responses and autoimmunity by repressing Tim-3–mediated cell death and exhaustion. Nat. Med..

[21] Tang R., Acharya N., Subramanian A., Purohit V., Tabaka M., Hou Y., He D., Dixon K.O., Lambden C., Xia J. (2022). Tim-3 adapter protein Bat3 acts as an endogenous regulator of tolerogenic dendritic cell function. Sci. Immunol..

[22] Zhu C., Dixon K.O., Newcomer K., Gu G., Xiao S., Zaghouani S., Schramm M.A., Wang C., Zhang H., Goto K. (2021). Tim-3 adaptor protein Bat3 is a molecular checkpoint of T cell terminal differentiation and exhaustion. Sci. Adv..

[23] Dixon K.O., Lahore G.F., Kuchroo V.K. (2024). Beyond T cell exhaustion: TIM-3 regulation of myeloid cells. Sci. Immunol..

[24] Alvisi G., Brummelman J., Puccio S., Mazza E.M.C., Tomada E.P., Losurdo A., Zanon V., Peano C., Colombo F.S., Scarpa A. (2020). IRF4 instructs effector Treg differentiation and immune suppression in human cancer. J. Clin. Investig..

[25] Yan H., Dai Y., Zhang X., Zhang H., Xiao X., Fu J., Zou D., Yu A., Jiang T., Li X.C. (2023). The transcription factor IRF4 determines the anti-tumor immunity of CD8+ T cells. iScience.

[26] Goldman M.J., Craft B., Hastie M., Repečka K., McDade F., Kamath A., Banerjee A., Luo Y., Rogers D., Brooks A.N. (2020). Visualizing and interpreting cancer genomics data via the Xena platform. Nat. Biotechnol..

[27] Cancer Genome Atlas Research N. (2008). Comprehensive genomic characterization defines human glioblastoma genes and core pathways. Nature.

[28] Brennan C.W., Verhaak R.G.W., McKenna A., Campos B., Noushmehr H., Salama S.R., Zheng S., Chakravarty D., Sanborn J.Z., Berman S.H. (2013). The Somatic Genomic Landscape of Glioblastoma. Cell.

[29] Neftel C., Laffy J., Filbin M.G., Hara T., Shore M.E., Rahme G.J., Richman A.R., Silverbush D., Shaw M.L., Hebert C.M. (2019). An Integrative Model of Cellular States, Plasticity, and Genetics for Glioblastoma. Cell.

[30] Blaser C., Kaufmann M., Pircher H. (1998). Cutting Edge: Virus-Activated CD8 T Cells and Lymphokine-Activated NK Cells Express the Mast Cell Function-Associated Antigen, An Inhibitory C-Type Lectin. J. Immunol..

[31] Chan C.J., Martinet L., Gilfillan S., Souza-Fonseca-Guimaraes F., Chow M.T., Town L., Ritchie D.S., Colonna M., Andrews D.M., Smyth M.J. (2014). The receptors CD96 and CD226 oppose each other in the regulation of natural killer cell functions. Nat. Immunol..

[32] Woroniecka K., Chongsathidkiet P., Rhodin K., Kemeny H., Dechant C., Farber S.H., Elsamadicy A.A., Cui X., Koyama S., Jackson C. (2018). T-Cell Exhaustion Signatures Vary with Tumor Type and Are Severe in Glioblastoma. Clin. Cancer Res..

[33] Borrego F., Robertson M.J., Ritz J., Peña J., Solana R. (1999). CD69 is a stimulatory receptor for natural killer cell and its cytotoxic effect is blocked by CD94 inhibitory receptor. Immunology.

[34] Cambiaggi C., Scupoli M.T., Cestari T., Gerosa F., Carra G., Tridente G., Accolla R.S. (1992). Constitutive expression of CD69 in interspecies T-cell hybrids and locus assignment to human chromosome 12. Immunogenetics.

[35] A Sabatos C., Chakravarti S., Cha E., Schubart A., Sánchez-Fueyo A., Zheng X.X., Coyle A.J., Strom T.B., Freeman G.J., Kuchroo V.K. (2003). Interaction of Tim-3 and Tim-3 ligand regulates T helper type 1 responses and induction of peripheral tolerance. Nat. Immunol..

[36] Sánchez-Fueyo A., Tian J., Picarella D., Domenig C., Zheng X.X., A Sabatos C., Manlongat N., Bender O., Kamradt T., Kuchroo V.K. (2003). Tim-3 inhibits T helper type 1–mediated auto- and alloimmune responses and promotes immunological tolerance. Nat. Immunol..

[37] Fu W., Wang W., Li H., Jiao Y., Huo R., Yan Z., Wang J., Wang S., Wang J., Chen D. (2020). Single-Cell Atlas Reveals Complexity of the Immunosuppressive Microenvironment of Initial and Recurrent Glioblastoma. Front. Immunol..

[38] Liu Z., Han H., He X., Li S., Wu C., Yu C., Wang S. (2016). Expression of the galectin-9-Tim-3 pathway in glioma tissues is associated with the clinical manifestations of glioma. Oncol. Lett..

[39] Mohme M., Schliffke S., Maire C.L., Rünger A., Glau L., Mende K.C., Matschke J., Gehbauer C., Akyüz N., Zapf S. (2018). Immunophenotyping of Newly Diagnosed and Recurrent Glioblastoma Defines Distinct Immune Exhaustion Profiles in Peripheral and Tumor-infiltrating Lymphocytes. Clin. Cancer Res..

[40] da Silva I.P., Gallois A., Jimenez-Baranda S., Khan S., Anderson A.C., Kuchroo V.K., Osman I., Bhardwaj N. (2014). Reversal of NK-Cell Exhaustion in Advanced Melanoma by Tim-3 Blockade. Cancer Immunol. Res..

[41] Xu L., Huang Y., Tan L., Yu W., Chen D., Lu C., He J., Wu G., Liu X., Zhang Y. (2015). Increased Tim-3 expression in peripheral NK cells predicts a poorer prognosis and Tim-3 blockade improves NK cell-mediated cytotoxicity in human lung adenocarcinoma. Int. Immunopharmacol..

[42] Rakova J., Truxova I., Holicek P., Salek C., Hensler M., Kasikova L., Pasulka J., Holubova M., Kovar M., Lysak D. (2021). TIM-3 levels correlate with enhanced NK cell cytotoxicity and improved clinical outcome in AML patients. OncoImmunology.

[43] Yuan F., Ming H., Wang Y., Yang Y., Yi L., Li T., Ma H., Tong L., Zhang L., Liu P. (2019). Molecular and clinical characterization of Galectin-9 in glioma through 1,027 samples. J. Cell. Physiol..

[44] Sim J., Park J., Kim S., Hwang S., Sung K., Lee J.-E., Yang S., Cho K., Lee S., Moon J.-S. (2022). Association of Tim-3/Gal-9 Axis with NLRC4 Inflammasome in Glioma Malignancy: Tim-3/Gal-9 Induce the NLRC4 Inflammasome. Int. J. Mol. Sci..

[45] Lim M., Weller M., Idbaih A., Steinbach J., Finocchiaro G., Raval R.R., Ansstas G., Baehring J., Taylor J.W., Honnorat J. (2022). Phase III trial of chemoradiotherapy with temozolomide plus nivolumab or placebo for newly diagnosed glioblastoma with methylated *MGMT* promoter. Neuro-Oncology.

