# Supplementary material for: Reduced T and NK Cell Activity in Glioblastoma Patients Correlates with TIM-3 and BAT3 Dysregulation
Source: Cells. 2024 Oct 26;13(21):1777. doi: 10.3390/cells13211777 (PMC11545661; doi:10.3390/cells13211777)
Supplement: Supplementary file 1 [file cells-13-01777-s001.zip › cells-3195342-supplementary.pdf]

Supplementary Materials

Reduced T and NK Cell Activity in Glioblastoma Patients Correlates with TIM-3 and BAT3 Dysregulation.

**Farah Ahmady[1,2], Peter Curpen[3], Louis Perriman[1,2,4], Adilson Fonseca Teixeira[5,6], Siqi Wu[5,6], Hong-Jian Zhu[5,6], Arpita Poddar[1,2], Aparna Jayachandran[1,2], George Kannourakis[1,2] and Rodney B. Luwor[1,2,5,6]*

[1]*Fiona Elsey Cancer Research Institute, Ballarat, Victoria 3350, Australia. Farah@fecri.org.au (F.A.); Arpita@fecri.org.au (A.P); Aparna@fecri.org.au (A.J.); George@fecri.org.au (G.K.); rodney@fecri.org.au (R.B.L.)*
[2]*Federation University, Ballarat, Victoria 3350, Australia.*
[3]*Townsville Hospital and Health Service, James Cook University, Townsville, Queensland 4814, Australia. JensenCurpen@outlook.com (P.C.)*
[4]*Murdoch Children's Research Institute, Parkville, Victoria 3052, Australia. Louis.perriman@mcri.edu.au (L.P.)*
[5]*Department of Surgery, The University of Melbourne, The Royal Melbourne Hospital, Parkville, Victoria 3050, Australia. afonsecateix@student.unimelb.edu.au (A.F.T.); Wus8@student.unimelb.edu.au (S.W.); hongjian@unimelb.edu.au (H-J.Z.)*
[6]*Huagene Institute, Kecheng Science and Technology Park, Pukou District, Nanjing 211806, Jiangsu, China*

* Correspondence: E-mail: rodney@fecri.org.au; Tel: +61 3 5331 3101; SMB Campus, Federation University Australia, Ballarat Central, Victoria 3350, Australia. ORCID Number: 0000-0002-3020-4245.

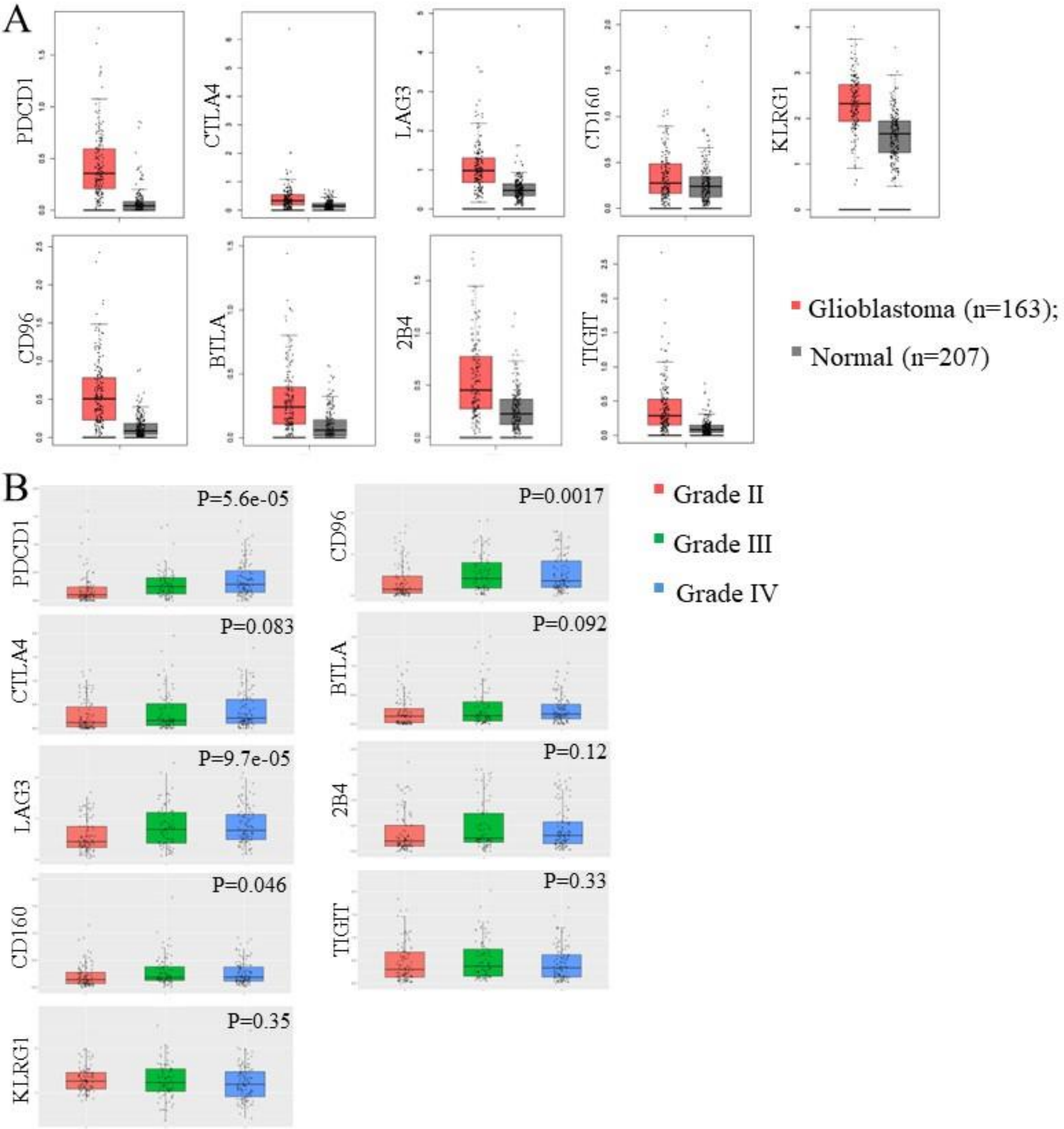
A
PDCD1
CTLA4
LAG3
CD160
KLRG1
CD96
BTLA
2B4
TIGIT
Glioblastoma (n=163);
Normal (n=207)
B
P=5.6e-05
P=0.0017
Grade II
Grade III
Grade IV
P=0.083
P=0.092
P=9.7e-05
P=0.12
P=0.046
P=0.33
P=0.35

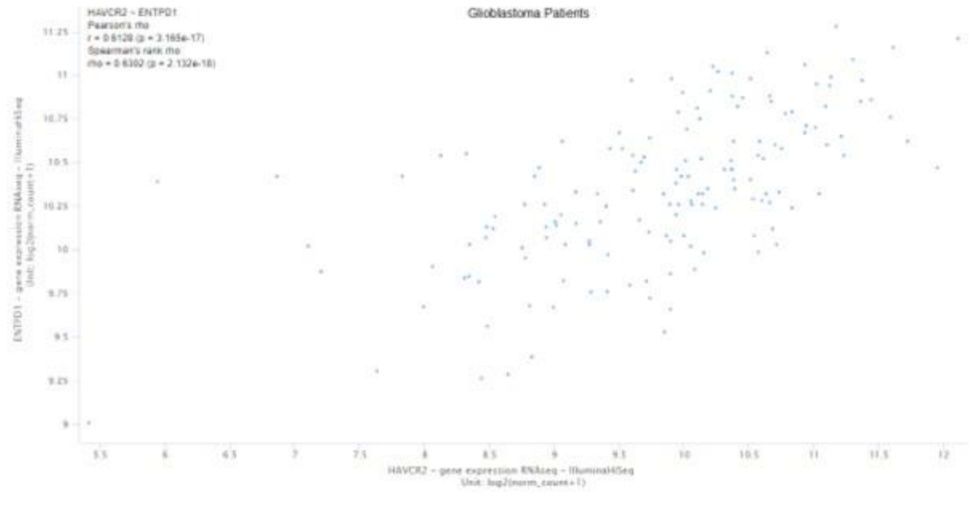
Glioblastoma Patients
HAVCR2 - ENTPD1
Pearson's rho
Spearman's rank rho

D

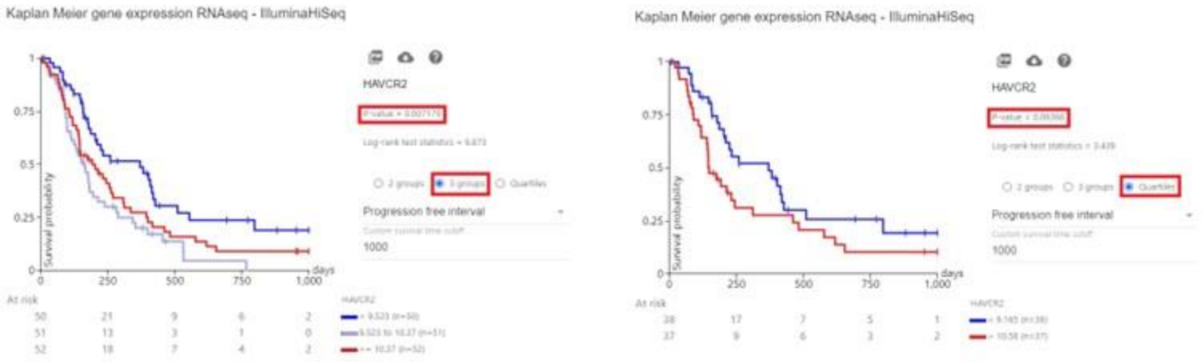

E

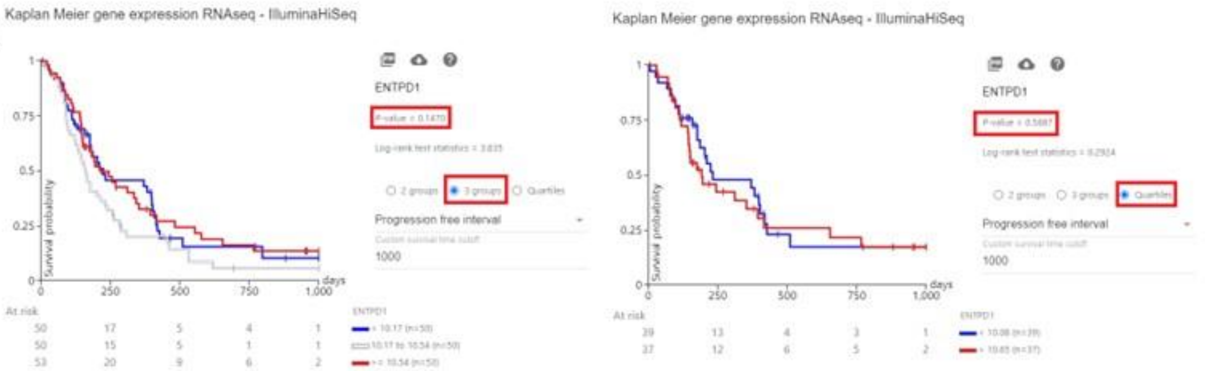

Suppl Figure 1: The Expression of Several Inhibitory or Checkpoint Receptors in Glioblastoma A. *PDCD1, CTLA4, LAG3, CD160, KLRG1, CD96, BTLA, 2B4 and TIGIT gene expression levels comparing glioblastoma tumor tissue (red; n = 163) and normal brain tissue low (grey; n = 207) were determined through mining the GEPIA2 TCGA dataset.* ***B.*** *PDCD1, CTLA4, LAG3, CD160, KLRG1, CD96, BTLA, 2B4 and TIGIT gene expression levels comparing glioblastoma (grade IV) tumor tissue (blue), grade III glioma tumor tissue (green) and grade II glioma tumor tissue (red) were determined through mining the CGGA dataset.* ***C.*** *Correlation between HAVCR2 and ENTPD1 mRNA levels in glioblastoma samples. Correlation of* ***D.*** *HAVCR2 and* ***E.*** *ENTPD1 mRNA levels when stratified into 3 groups or when comparing first and fourth quartiles.*

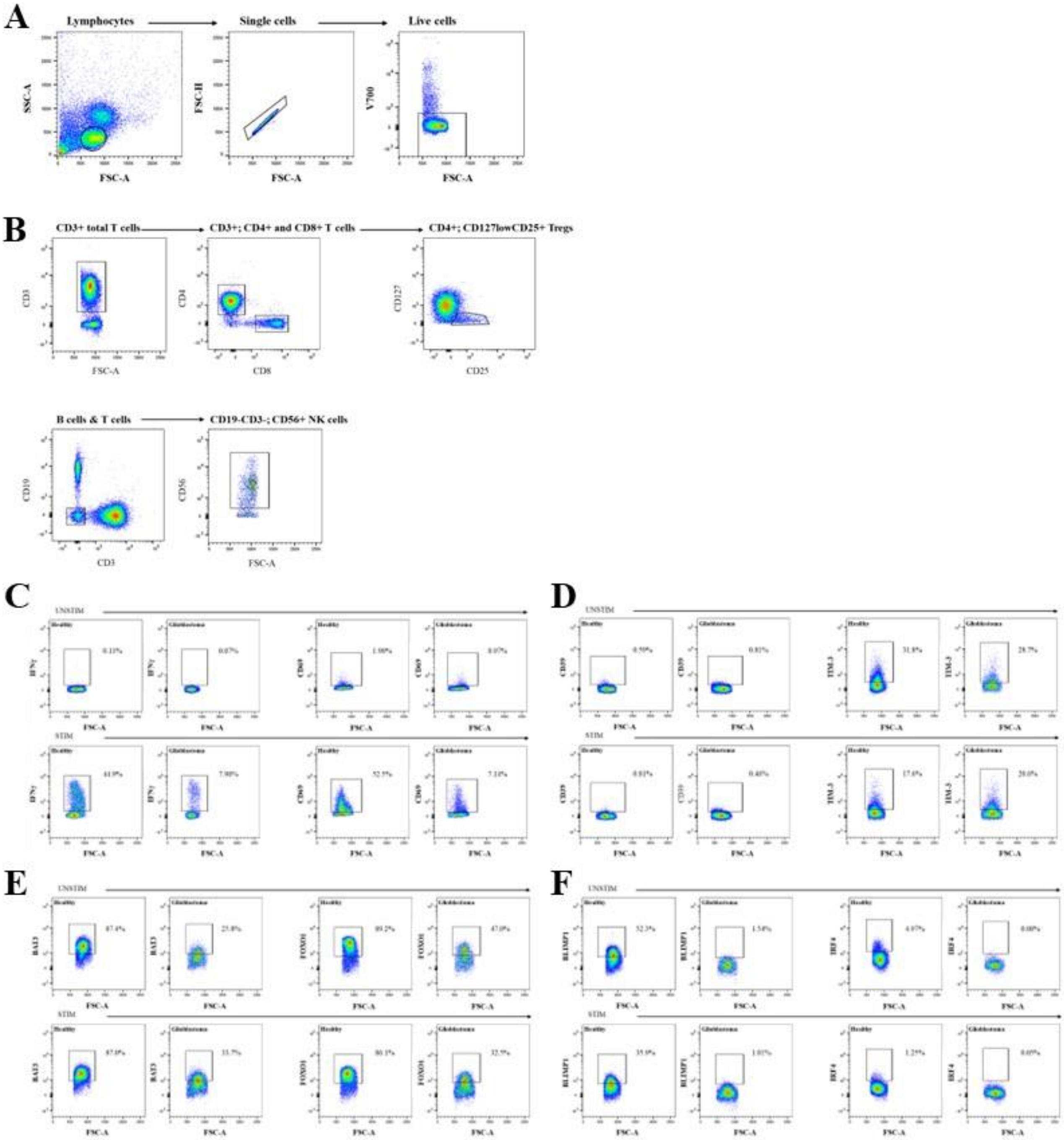

Suppl Figure 2: Gating Strategy for Flow Cytometry of PBMCs from Healthy Donors and Glioblastoma Patients. A. *Debris were excluded, and lymphocytes included, using a forward scatter area (FSC-A) (size) versus side scatter area (SSC-A) (granularity) gate. Single cells (singlets) were then selected on an FSC-A versus forward scatter height (FSC-H) plot to exclude doublets and live cells were selected on a V700 versus FSC-A plot.* ***B.*** *T cell subsets were selected using CD3^{+} versus FSC-A; CD4^{+} versus CD8^{+} and CD127^{low} versus CD25^{+} gate. NK cells were selected using a CD19^{-} versus CD3^{-}; CD56^{+} gate. Similarly, gating strategy for* ***C.*** *IFNγ and CD69;* ***D.*** *CD39 and TIM-3;* ***E.*** *BAT3 and FOXO1 and* ***F.*** *BLIMP1 and IRF4 for unstimulated and stimulated healthy and glioblastoma cells.*

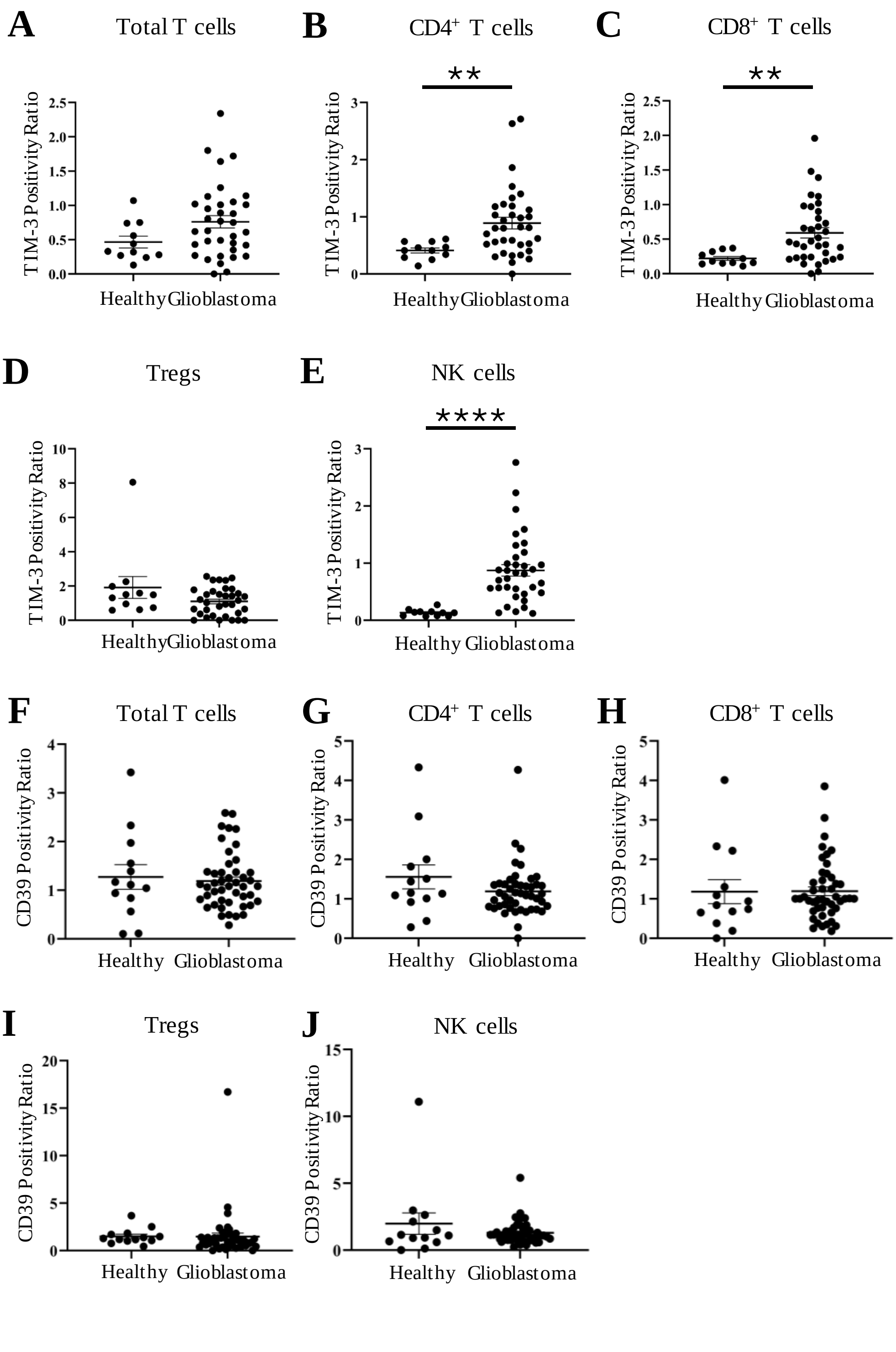

Suppl Fig 3. Percentage of TIM-3 Positivity is Maintained after Stimulation on CD4⁺ T, CD8⁺ T and NK cells from Glioblastoma Patients. PBMCs from healthy controls and glioblastoma patients were stimulated with PMA and ionomycin for 5 hours, then stained for surface TIM-3 and CD39 for flow cytometry analysis. The ratio of unstimulated percentage of TIM-3 positive to stimulated percentage of TIM-3 positive are presented for each healthy control (n = 11) and glioblastoma patient (n = 34-35) for A. total T cells, B. $CD4^+$ T cells, C. $CD8^+$ T cells, D. Tregs and E. NK cells. The ratio of unstimulated percentage of CD39 positive to stimulated percentage of CD39 positive are presented for each healthy

control (n = 13) and glioblastoma patient (n = 46) for F. total T cells, G. CD4+ T cells, H. CD8+ T cells, I. Tregs and J. NK cells. **p ≤ 0.01, ****p ≤ 0.0001.

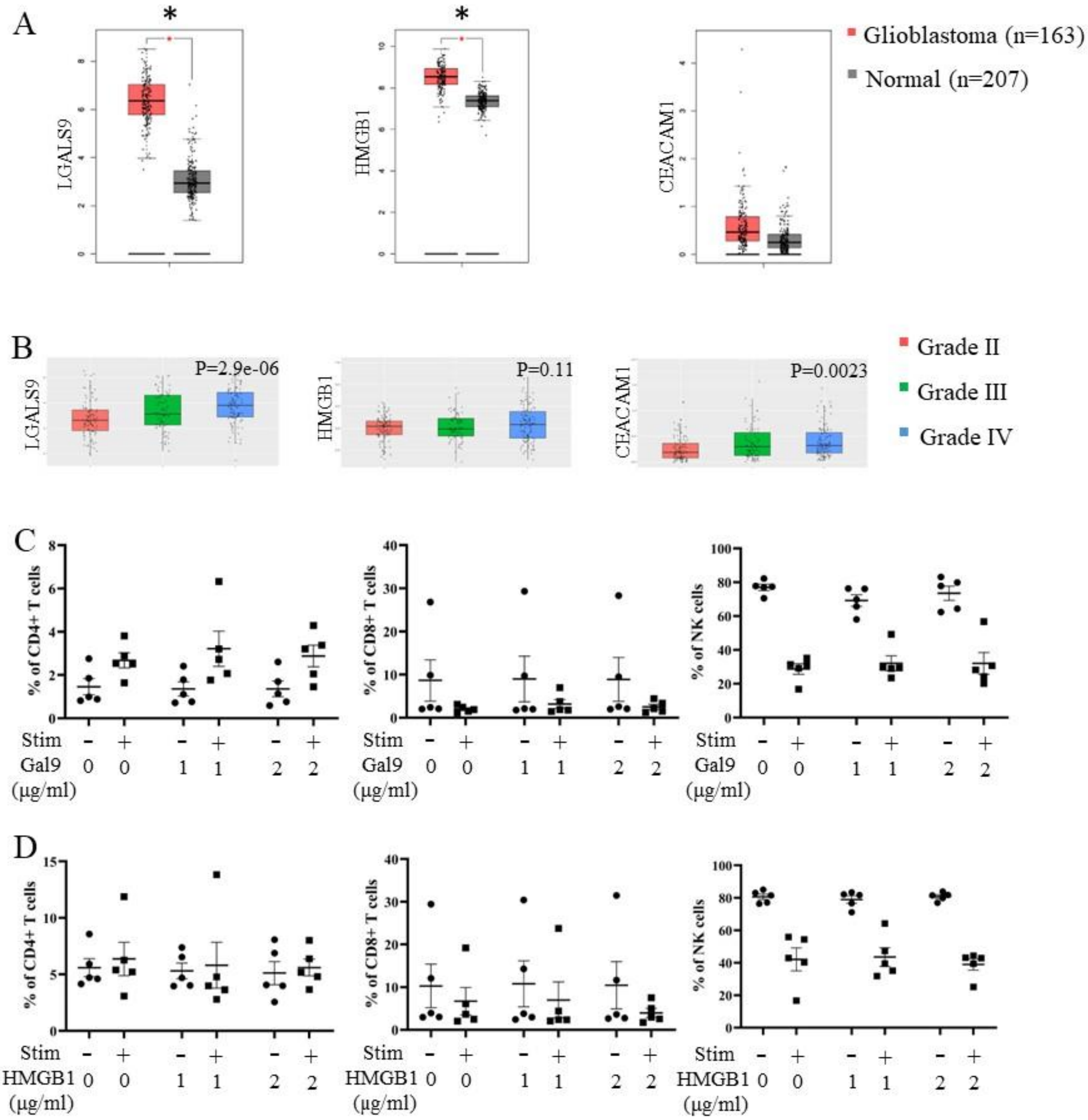

Suppl Figure 4: LGALS and HMGB1 are over-expressed in Glioblastoma but do not Change Percentage of TIM-3 Positivity. ***A.*** *LGALS9, HMGB1 and CEACAM1 gene expression levels comparing glioblastoma tumor tissue (red; n = 163) and normal brain tissue low (grey; n = 207) were determined through mining the GEPIA2 TCGA dataset.* ***B.*** *LGALS9, HMGB1 and CEACAM1 gene expression levels comparing glioblastoma (grade IV) tumor tissue (blue), grade III glioma tumor*

*tissue (green) and grade II glioma tumor tissue (red) were determined through mining the CGGA dataset. Healthy donor PBMCs (n = 5) were treated with 0, 1 and 2 µg/ml of **C.** galectin-9 or **D.** HMGB1 for 24 hours, stimulated with PMA, ionomycin and Golgiplug™ for 5 hours, then stained for surface TIM-3 for flow cytometry analysis. The unstimulated and stimulated percentage of TIM-3 positivity for each condition are presented. *p ≤ 0.05.*

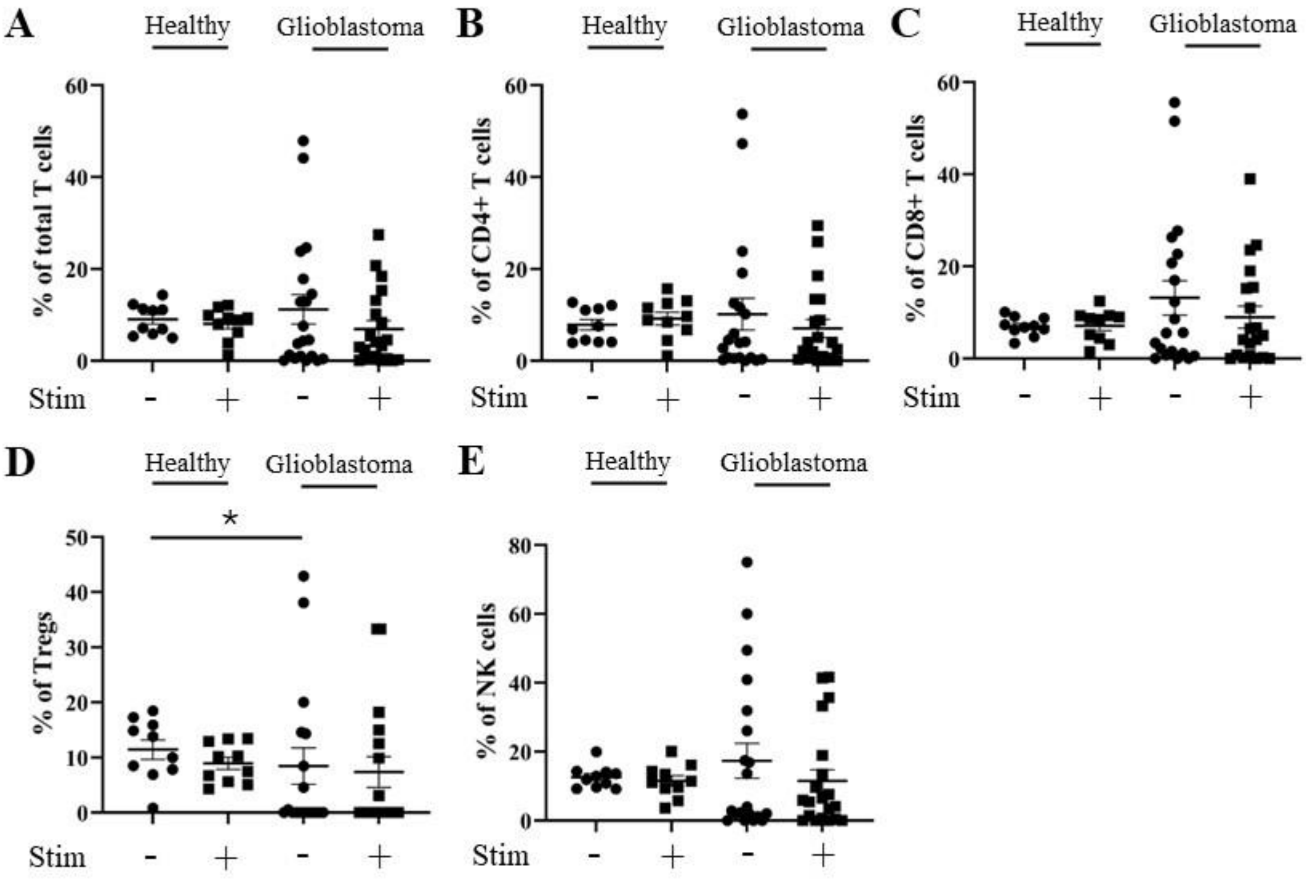

Suppl Figure 5: Percentage of IRF4 Positivity is Similar on CD4⁺ T, CD8⁺ T and NK cells from Healthy Control Donors and Glioblastoma Patients. *PBMCs from healthy controls and glioblastoma patients were treated with PMA and ionomycin and Golgiplug™ for 5 hours, then stained for intracellular IRF4 for flow cytometry analysis. The unstimulated and stimulated percentage of IRF4 positivity for each healthy control (n = 10) and glioblastoma patient (n = 20) are presented for **A.** total T cells, **B.** CD4⁺ T cells, **C.** CD8⁺ T cells, **D.** Tregs and **E.** NK cells. *p ≤ 0.05.*

Supplementary Table 1: Healthy donor and Glioblastoma Patient Characteristics

	**Healthy Donors**	**Glioblastoma Patients**
**N**	17	46
**Gender (F:M %)**	29:71	22:78
**Age ± S.D. (years)**	55 ± 12.7	65 ± 10.1

**Age Range (years)**	35 – 74	40 - 85
**Survival[a] ± S.D. (days)**	N/A	372 ± 358
**Survival Range (days)**	N/A	32 - 2015

[a]Survival is calculated as number of days from date of diagnosis of primary glioblastoma to date of death.
